# Shut-Down of Type IX Protein Secretion Alters the Host Immune Response to *Tannerella forsythia* and *Porphyromonas gingivalis*

**DOI:** 10.3389/fcimb.2022.835509

**Published:** 2022-02-10

**Authors:** Matthias L. Braun, Markus B. Tomek, Clemens Grünwald-Gruber, Phuong Q. Nguyen, Susanne Bloch, Jan S. Potempa, Oleh Andrukhov, Christina Schäffer

**Affiliations:** ^1^ Department of NanoBiotechnology, NanoGlycobiology Unit, Universität für Bodenkultur Wien, Vienna, Austria; ^2^ Department of Chemistry, Institute of Biochemistry, Universität für Bodenkultur Wien, Vienna, Austria; ^3^ Competence Center for Periodontal Research, University Clinic of Dentistry, Medical University of Vienna, Vienna, Austria; ^4^ Oral Health and Systemic Disease Group, University of Louisville, Louisville, KY, United States

**Keywords:** antibacterial target, periodontitis, periodontal pathogen, protein secretion, virulence factor

## Abstract

*Tannerella forsythia* and *Porphyromonas gingivalis* target distinct virulence factors bearing a structurally conserved C-terminal domain (CTD) to the type IX protein secretion system (T9SS). The T9SS comprises an outer membrane translocation complex which works in concert with a signal peptidase for CTD cleavage. Among prominent T9SS cargo linked to periodontal diseases are the TfsA and TfsB components of *T. forsythia’s* cell surface (S-) layer, the bacterium’s BspA surface antigen and a set of cysteine proteinases (gingipains) from *P. gingivalis*. To assess the overall role of the bacterial T9SS in the host response, human macrophages and human gingival fibroblasts were stimulated with *T. forsythia* and *P. gingivalis* wild-type bacteria and T9SS signal peptidase-deficient mutants defective in protein secretion, respectively. The immunostimulatory potential of these bacteria was compared by analyzing the mRNA expression levels of the pro-inflammatory mediators IL-6, IL-8, MCP-1 and TNF-α by qPCR and by measuring the production of the corresponding proteins by ELISA. Shot-gun proteomics analysis of *T. forsythia* and *P. gingivalis* outer membrane preparations confirmed that several CTD-bearing virulence factors which interact with the human immune system were depleted from the signal peptidase mutants, supportive of effective T9SS shut-down. Three and, more profoundly, 16 hours post stimulation, the *T. forsythia* T9SS mutant induced significantly less production of cytokines and the chemokine in human cells compared to the corresponding parent strain, while the opposite was observed for the *P. gingivalis* T9SS mutant. Our data indicate that T9SS shut-down translates into an altered inflammatory response in periodontal pathogens. Thus, the T9SS as a potential novel target for periodontal therapy needs further evaluation.

## Introduction

Oral health is characterized by the symbiotic interaction between the oral microbiota and the human host. Its disturbance by environmental or genetic factors leads to microbial dysbiosis and increases the risk of oral diseases, particularly periodontitis (Marsh and Zaura, 2017). Periodontitis is an inflammatory biofilm disease of the tooth-supporting tissues characterized by a dysbiotic state and the prevalence of the “red complex” of Gram-negative, anaerobic pathogens-*Porphyromonas gingivalis, Tannerella forsythia* and *Treponema denticola* (Holt and Ebersole, 2005; Socransky and Haffajee, 2005; Griffen et al., 2012). One of these bacteria, *P. gingivalis*, is considered as a keystone pathogen and can subvert the host immune response, disrupting the host-microbe homeostasis in the oral cavity and promoting a dysbiotic state, even when present at low quantities (Hajishengallis and Lamont, 2012).

The “red complex” bacteria interfere with metabolic and physiological functions of the host through virulence factors (Lasica et al., 2017; Gorasia et al., 2020). *P. gingivalis* and *T. forsythia* secrete distinct virulence factors across the outer membrane (OM) using the type IX secretion system (T9SS), which is regarded as an essential determinant of pathogenicity in periodontal diseases (Tomek et al., 2014). The T9SS seems to be characteristic of the *Fibrobacteres*–*Chlorobi*–*Bacteroidetes* superphylum to which *T. forsythia* and *P. gingivalis* are affiliated (Lasica et al., 2017). The T9SS machinery is composed of at least 18 essential protein components, of which orthologs exist in *P. gingivalis* and *T. forsythia* (Lasica et al., 2017; Lauber et al., 2018). These components build up a complex that translocates proteins possessing a structurally conserved carboxy-terminal domain (CTD) *via* the OM. The “classical” CTD is composed of 40-70 variable amino acid residues that possess an Ig-like fold (de Diego et al., 2016). The 3D structure of the CTD serves as a recognition element of proteins for the T9SS. After protein translocation to the surface, CTD-cleavage is catalyzed by a C-terminal signal peptidase named PG0026 (PorU) and Tanf_02580 in *P. gingivalis* W83 and *T. forsythia* ATCC 43037, respectively (Tomek et al., 2014; Lasica et al., 2016), components of the attachment complex built of PorQ, PorU, PorV and PorZ (Nguyen et al., 2007; Veith et al., 2009; Sato et al., 2013; Lauber et al., 2018). T9SS cargo proteins are either released to the environment (Lasica et al., 2017) or stay associated with the bacterial surface, predictably anchored into the OM by a glycoconjugate of so far unknown structure that is attached to the C-terminal residue (Veith et al., 2020).

In *P. gingivalis*, the gingipains-RgpA, RgpB, and Kgp-are intensely investigated cysteine proteases carrying a “classical” CTD for secretion *via* the T9SS; they have a myriad of roles in periodontitis. Gingipains cause hypo-responsiveness of several components of innate immunity like epithelial cells, macrophages, and neutrophils (Stathopoulou et al., 2009; Wilensky et al., 2015; Sochalska and Potempa, 2017), resulting in impaired bacterial clearance and a dysbiotic state. Furthermore, they degrade host cytokines and chemokines resulting in downregulation of the host response in the form of reduced inflammation (Stathopoulou et al., 2009; Stathopoulou et al., 2010). While gingipains are mainly attached to the surface of the OM, they may also be partially released in a soluble form into the extracellular milieu (Rangarajan et al., 1997). It was shown that a C-terminally truncated form of RgpB is no longer attached to the OM demonstrating the importance of the CTD signal for export and cell attachment (Seers et al., 2006).

Among prominent virulence proteins of *T. forsythia* which are equipped with a “classical” CTD for targeting to the T9SS are, for instance, the two heavily glycosylated surface (S-) layer proteins TfsA and TfsB (Sabet et al., 2003; Lee et al., 2006; Sakakibara et al., 2007; Sekot et al., 2011; Posch et al., 2012), BspA surface antigen (Sharma et al., 1998), and hemagglutinin (Murakami et al., 2002). The TfsA and TfsB proteins self-assemble into a 2D crystalline layer around *T. forsythia* cells; this S-layer mediates the adherence of the bacterium to the human gingival epithelium (Mishima and Sharma, 2011) and, at the early stage of infection, delays the immune response of human gingival fibroblasts (hGFBs) and macrophages (Sekot et al., 2011). BspA, on the other hand, is known to activate the host response in monocytes and epithelial cells through a TLR-2 dependent mechanism (Hajishengallis et al., 2002; Onishi et al., 2008). Furthermore, a set of six secretory proteases of *T. forsythia* cleave host proteins such as collagen (Ksiazek et al., 2015) and degrade complement proteins and the antimicrobial peptide LL-37, which may contribute to virulence through evading innate immunity (Koneru et al., 2017). Of note, these proteases bear a nearly identical CTD that ends with a Lys-Leu-Ile-Lys-Lys motif (KLIKK), but share very limited sequence similarity with the “classical” CTD (Ksiazek et al., 2015).

Considering the link between CTD-proteins and virulence, it is likely that blocking of their OM-export in *T. forsythia* and *P. gingivalis* alters the pathogens’ ability to induce a host response. Thus, the purpose of this study was to assess, if and in which aspects shut-down of the T9SS in *T. forsythia* and *P. gingivalis* influences the elicitation of a cellular response of human macrophages and hGFBs-both known for their roles in the pathogenesis of periodontitis. Specifically, along with the parent strains we used mutants of *T. forsythia* and *P. gingivalis* with a deletion in the T9SS signal peptidase gene resulting in a secretion defective phenotype. Using shot-gun proteomics, we first investigated the bacterias’ OM proteome for the presence of key CTD-proteins of known virulence potential to assess the efficiency of the T9SS shut-down and then, challenged the two human cell types with the different bacterial species for up to 16 hours and determined the production of different inflammatory mediators using qPCR and ELISA. Specifically, we investigated the production of tumor necrosis factor (TNF)-α, interleukin (IL)-8, and IL-6 in U937 macrophages and of IL-6, IL-8, and monocyte chemoattractant protein (MCP)-1 in hGFBs. IL-6 and TNF-α are involved in regulating the immune reactivity of acute-phase proteins and in recruiting of lymphocytes to inflamed tissues (Charlie-Silva et al., 2019). IL-8 and MCP-1 are strong chemoattractants and stimulate the migration of leukocytes to sites of infection (Baggiolini and Clark-Lewis, 1992; Deshmane et al., 2009). These inflammatory mediators are a substantial part of the host defense; insufficient cytokine production leads to an impaired bacterial elimination and development of a dysbiotic state (Olsen and Hajishengallis, 2016), whereas an excessive response might cause collateral tissue damages (Cekici et al., 2014).

## Materials and Methods

### Bacterial Strains and Growth Conditions

The *T. forsythia* ATCC 43037 type strain (wild-type) and *P. gingivalis* BAA-308/W83 (wild-type) were obtained from the American Type Culture Collection (ATCC, Manassas, VA, USA). Deletion mutants of the signal peptidase gene encoding PorU (*PG0026*) of *P. gingivalis* (Lasica et al., 2016) (henceforth abbreviated Δ*PG0026*) and the *T. forsythia* ortholog *Tanf_02580* (henceforth abbreviated Δ*Tanf_02580*) (Tomek et al., 2014) were available in our laboratory. *T. forsythia* wild-type and Δ*Tanf_02580* were grown anaerobically in brain-heart-infusion (BHI) broth with supplements, at 37°C for 7 days as published previously (Tomek et al., 2014). *P. gingivalis* wild-type and Δ*PG0026* were grown anaerobically in enriched tryptic soy broth (ETSB) at 37°C for 3 days (Tada et al., 2017). Bacteria were harvested by centrifugation at 5000 *g* for 20 min at 4°C and washed once with the respective growth medium.

For stimulation of human cells, bacterial pellets were resuspended in RPMI 1640 medium (Invitrogen, Waltham, MA, USA) and the optical density at 600 nm (OD_600_) was set to 1 with medium. A correlation between OD_600_ values of 1.0 and colony forming units (CFU) per milliliter of culture of the different bacteria and mutants included in this study was determined by dilution plating and colony counting (three biological replicates with three technical replicates, each), with OD_600_ = 1.0 corresponding to 3 x 10^8^ CFU of *T. forsythia* wild-type, 5 x 10^8^ CFU of *T. forsythia* Δ*Tanf_02580*, 1 x 10^9^ CFU of *P. gingivalis* wild-type, and 1 x 10^9^ CFU of *P. gingivalis* Δ*PG0026*, respectively. Bacterial suspensions were immediately used for further processing.

### Outer Membrane Isolation

For the isolation of outer membranes, 1 g of wet pellet of *T. forsythia* wild-type, Δ*Tanf_02580*, *P. gingivalis* wild-type, and Δ*PG0026*, respectively, was resuspended in 25 mL of phosphate-buffered saline (PBS) and disrupted by sonication on ice for 15 min/50% power/30 s duty cycle, using a Branson Ultrasonics Sonifier™ (Branson, Brookfield, CT, USA). The OM isolation essentially followed a protocol published for *T. forsythia* (Komatsuzawa et al., 2002; Shimotahira et al., 2013). Briefly, undisrupted bacteria were removed and membrane fractions collected by ultracentrifugation at 100,000 *g* for 1 h/4°C (Ti70.1 rotor; Beckman, Brea, CA, USA) and washed with PBS. The pellet was resuspended in 5 mL of 1% (*w/v*) sodium lauryl sarcosine (Sigma-Aldrich, St. Louis, MO, USA) and the insoluble OM fraction was re-dissolved in 500 µL of 1% (*w/v*) sodium dodecyl sulphate (SDS) (*w/v*). The OM isolation was performed in triplicate, each, and the preparations were analyzed by SDS-PAGE using 10% gels according to Laemmli (Laemmli, 1970) and separated protein bands were stained with Coomassie Brilliant Blue G250 (CBB). Protein concentrations of the OM preparations were determined by the Bradford assay (Bradford, 1976).

### Shot-Gun Proteomics

For shot-gun proteomics, the SDS-dissolved OM preparations were digested in-solution. The proteins were S-alkylated with iodoacetamide and digested with trypsin (Promega, Madison, WI, USA) as described elsewhere (Gundry et al., 2009). The digested samples were loaded on a BioBasic C18 column (BioBasic-18, 150 x 0.32 mm, 5 µm; Thermo Fisher Scientific, Waltham, MA, USA) using 80 mM ammonium formate buffer at a flow rate of 6 µL min^-1^ as solvent A. A gradient from 5% solvent B (80% acetonitrile) in solvent A to 40% solvent B over 45 min was applied, followed by a 15-min gradient from 40% solvent B to 95% solvent B to facilitate the elution of large peptides. Detection was performed with QTOF MS (Bruker maXis 4G; Bruker, Billerica, MA, USA) equipped with the standard ESI source in positive ion, DDA mode (switching to MSMS mode for eluting peaks). MS-scans were recorded (range: 150-2200 Da) and the six highest peaks were selected for fragmentation. For instrument calibration, ESI calibration mixture (Agilent, Santa Clara, CA, USA) was used. The analysis files were converted to mgf files (using Data Analysis, Bruker), which are suitable for performing an MS/MS ion search with ProteinScape (Bruker, MASCOT embedded). The files were searched against the UniProt database.

### Isolation and Growth Conditions of Human Monocytes and Human Gingival Fibroblasts

The U937 monocytic cell line was purchased from the ATCC and cultured in RPMI 1640 medium, supplemented with 10% (*v/v*) fetal bovine serum (FBS) and penicillin (100 U mL^-1^)-streptomycin (100 µg mL^-1^) (Pen-Strep) at 37°C in a humidified atmosphere containing 5% CO_2_ (Friedrich et al., 2015).

hGFBs were isolated from the gingival tissue of periodontally and systemically healthy individuals (Sekot et al., 2011). Gingival tissue was cut off with a scalpel, placed into Dulbecco’s Modified Eagle’s Medium (DMEM; Invitrogen) supplemented with 10% FBS, Pen-Strep, shredded into small pieces, and incubated at 37°C and 5% CO_2_ for cell outgrowth.

### Stimulation of Human Macrophages and Human Gingival Fibroblasts With Bacteria

Prior to stimulation with bacteria, U937 monocytes were differentiated into macrophages as described previously (Sekot et al., 2011; Friedrich et al., 2015). Briefly, three milliliters of cell suspension at a concentration of 10^6^ cells mL^-1^ were added per well of a 6-well plate and cells were stimulated with phorbol 12-myristate 13-acetate (Sigma-Aldrich) at a concentration of 0.2 μg mL^-1^ for 72 hours.

Adherent macrophages were gently scraped, counted and seeded in a 24-well plate at a density of 2 x10^5^ cells/well in 0.5 mL of RPMI 1640 medium supplemented with 10% FBS and 1% Pen-Strep. hGFBs were seeded at a density of 5 x10^4^ cells/well in 0.5 mL of DMEM containing the same supplements. After 24 hours, the media were discarded, cells were rinsed once with PBS, subsequently, 0.5 mL of the respective medium without FBS, but containing Pen-Strep was added, and cells were exposed to the different bacterial stimuli at a multiplicity of infection (MOI) of 50 (Sekot et al., 2011). Six and five independent experiments for U937 and hGFB, respectively, with four technical replicates, each, were performed and FBS-free medium containing Pen-Strep without bacteria served as a negative control. Stimulation was done for 3 hours and 16 hours, at 37°C and 5% CO_2_.

### MTT Cell Viability Assay

After cell stimulation, 100 µL of 3-(4,5-dimethylthiazol-2-yl)-2,5-diphenyltetrazolium bromide (MTT) dye (5 mg mL^-1^ in PBS) was added to the cells and the plates were incubated at 37°C for 2 hours (Vistica et al., 1991). Subsequently, the medium was discarded and 500 µL of dimethylsulfoxide were added to each well and the plates were shaken to facilitate dissolving of formazan crystals. Controls were performed in which each bacterium was solely added. OD_570_ values were measured on a Spectramax Plus micro-plate reader (Molecular Devices, Sunnyvale, CA, USA). The MTT assay was performed in five replicates.

### Gene Expression Analysis of Inflammatory Mediators

At the end of the stimulation, the cell supernatant was collected and aspirated for ELISA quantification of secreted inflammatory mediators. Adherent cells were washed with PBS followed by detachment from the wells with a cell scraper (macrophages) or accutase (hGFBs; Thermofisher Scientific) and used for gene-expression analysis.

Isolation of mRNA, transcription into cDNA, and qPCR was performed using the TaqMan^®^ Gene Expression Cells-to-CT™ kit (Ambion/Applied Biosystems, Foster City, CA, USA) (Behm et al., 2019; Blufstein et al., 2019). The target genes were amplified using the following primers (all Applied Biosystems): TNF-α, Hs99999043_m1; IL-6, Hs00985639_m1; IL-8, Hs00174103_m1; MCP-1, Hs00234140; GAPDH, Hs99999905_m1. qPCR was performed in paired reactions using the ABI StepOnePlus device with the following setting: 10 min at 95°C, 50 cycles at 95°C for 15 seconds and 60°C for 60 seconds. C_t_ values were determined for each gene and the expression of the target gene was determined by the 2^−ΔΔCt^ method, where $\Delta \Delta {\text{C}}_{\text{t}}=\left({\text{C}}_{\text{t}}^{\text{target}}-{\text{C}}_{\text{t}}^{\text{GAPDH}}\right)\text{sample}-({\text{C}}_{\text{t}}^{\text{target}}-{\text{C}}_{\text{t}}^{\text{GAPDH}})\text{control}$ . Cells, which were not treated with bacteria, served as control. For U937 macrophages, expression of IL-6, IL-8 and TNF-α was analyzed, for hGFBs, IL-6, IL-8 and MCP-1.

### Determination of Secreted Cytokines and Chemokines by ELISA

The concentration of the inflammatory mediators IL-6, IL-8, TNF-α, and MCP-1 in conditioned media, *i.e*., cell culture supernatant after stimulation, was determined using ELISA Ready-SET-Go kits (eBioscience, Santa Clara, CA, USA) according to the manufacturer’s protocol.

### Statistical Analysis

ANOVA’s statistic for the repeated measure followed by the post-hoc LSD test for pairwise comparisons was used to analyze statistical differences. Statistical analysis was performed using SPSS 24.0 software (IBM, Armonk, NY, USA). All data are expressed as mean ± standard error of the mean (s.e.m.). Significant statistical differences were considered at *P* < 0.05.

## Results

### CTD-Proteins Are Depleted From the Outer Membrane of a *T. forsythia* and *P. gingivalis* T9SS Signal Peptidase Mutant

Prior to analyzing the influence of the T9SS shut-down on the immune response of U937 macrophages and hGFBs to *T. forsythia* and *P. gingivalis* challenges, the efficiency of CTD-protein depletion from the OM in the signal peptidase mutants Δ*Tanf_02580* (Tomek et al., 2014) and Δ*PG0026* was investigated.

First, SDS-PAGE analysis of the OM preparations from *T. forsythia* wild-type, Δ*Tanf_02580*, *P. gingivalis* wild-type, and Δ*PG0026* revealed different CBB-stained banding patterns of the respective parent and mutant strains, indicative of the absence of proteins in the molecular-mass regions of known CTD-proteins (compare with **Tables 1**, **2**) in the T9SS signal peptidase mutants (**Supplementary Figure 1**).

**Table 1:** CTD proteins detected by MS in the OM from *T. forsythia* ATCC 43037 wild-type (wt *Tf*) and T9SS signal peptidase-deficient mutant (Δ*Tanf_02580*).

Protein name	Protein		MASCOT score*		Sequence coverage (%)**	
	Locus tag	Amino acids	wt *Tf*	Δ*Tanf_02580*	wt *Tf*	*ΔTanf_02580*
Surface antigen BspA	Tanf_04820	1156	784.7	0	19.1	0
Possible hemagglutinin	Tanf_06020	1252	134.3	0	5.2	0
S-layer protein TfsA	Tanf_03370	1166	4002	350	49.9	7.8
S-layer protein TfsB	Tanf_03375	1347	3415	0	46,7	0
IgG Fc binding domain-containing protein	Tanf_00065	598	1228	0	29.1	0
IgG Fc binding domain-containing protein	Tanf_11855	613	1235	0	24.3	0
Bacterial group 2 Ig-like protein	Tanf_03310	376	241.2	0	12	0
Conserved repeat protein	Tanf_08920	764	212.3	0	5.8	0
Hypothetical protein, uncharacterized	Tanf_08965	1562	670	0	9.6	0
Hypothetical protein, uncharacterized	Tanf_02330	1830	477.5	0	8.1	0
Hypothetical protein, uncharacterized	Tanf_02425	1457	976.4	0	19.4	0

*Results from one representative preparation from three biological replicates in terms of MASCOT scores are shown. **Only peptides covering more than 5% of the sequence were considered.

**Table 2:** CTD proteins detected by MS in the OM from *P. gingivalis* W83 wild-type (wt *Pg*) and T9SS signal peptidase-deficient mutant (Δ*PG0026*).

Protein name	Protein		MASCOT score*		Sequence coverage (%)**	
	Locus tag	Amino acids	wt *Pg*	Δ*PG0026*	wt *Pg*	Δ*PG0026*
Arginine-specific protease RgpA	PG2024	1706	2140	0	23	0
Arginine-specific cysteine proteinase RgpB	PG0506	736	915	0	27	0
Lysine-specific cysteine proteinase Kgp	PG1844	1727	2145	0	22	0
Hemagglutinin protein HagA	PG1837	2105	908	0	13	0
Immunoreactive 61-kDa antigen PG91	PG2102	540	332	0	13	0
Peptidyl-arginine deaminase	PG1424	446	804	0	27	0

*Results from one representative preparation from three biological replicates in terms of MASCOT scores are shown. **Only peptides covering more than 5% of the sequence were considered.

Next, the OM preparations from *T. forsythia* wild-type, Δ*Tanf_02580*, *P. gingivalis* wild-type, and Δ*PG0026* were subjected to shot-gun proteomics. Specifically, we performed a closer inspection for the presence of known CTD-proteins in order to assess the efficiency of their translocation blockage *via* deletion of the T9SS signal peptidase genes *PG0026* and *Tanf_02580*, respectively.

According to MS analysis of peptide fingerprints, in the OM preparation of the Δ*Tanf_02580* mutant (**Table 1**), several known CTD-bearing virulence factors were no longer detectable. Among these were the surface antigen BspA (Tanf_04820), a possible hemagglutinin/hemolysin (Tanf_06020) and the S-layer protein TfsB (Tanf_03375), while the second S-layer protein, TfsA (Tanf_03370) was massively reduced. It is likely that due to the high cellular abundance of the S-layer proteins residual amounts of TfsA might originate from cross-contamination with periplasmic content during cell fractionation. These results not only confirm but also expand our previous analysis of the Δ*Tanf_02580* mutant (Tomek et al., 2014) and corroborate data showing that other *T. forsythia* CTD-proteins (Veith et al., 2009) are missing in the OM fraction of the signal peptidase mutant but are present in that of the *T. forsythia* wild-type. These included IgG Fc binding domain-containing proteins (Tanf_00065, Tanf_11855), a bacterial group 2 Ig-like protein (Tanf_03310), a conserved repeat protein (Tanf_08920), as well as hypothetical proteins (Tanf_08965, Tanf_02330, Tanf_02425). Notably, in contrast to these proteins bearing a “classical” CTD, the KLIKK proteases of *T. forsythia* are secreted directly into the extracellular medium, and, thus, cannot be detected in either the wild-type bacterium or the T9SS signal peptidase mutant (Veith et al., 2009).

In the case of *P. gingivalis*, peptide fingerprints of the dominant *P. gingivalis* Cys-proteases RgpA (PG2024), RgpB (Arg-specific; PG0506) and Kgp (Lys-specific; PG1844) as well as of hemagglutinin HagA (PG1837), immunoreactive antigen PG91 (PG2102) and peptidylarginine deiminase (PG1424) were found exclusively in the OM preparation form the *P. gingivalis* wild-type (**Table 2**).

### Effect of *T. forsythia* and *P. gingivalis* Wild-Type and the Δ*Tanf_02580* and Δ*PG0026* Mutant on the Viability of U937 Macrophages and Gingival Fibroblasts

The effect of the different wild-type bacteria and mutants on the viability of U937 macrophages and hGFBs was investigated prior to determining the immunostimulatory potential of *T. forsythia* and *P. gingivalis* wild-type *versus* the respective T9SS signal peptidase deficient mutant.

**Figure 1:**
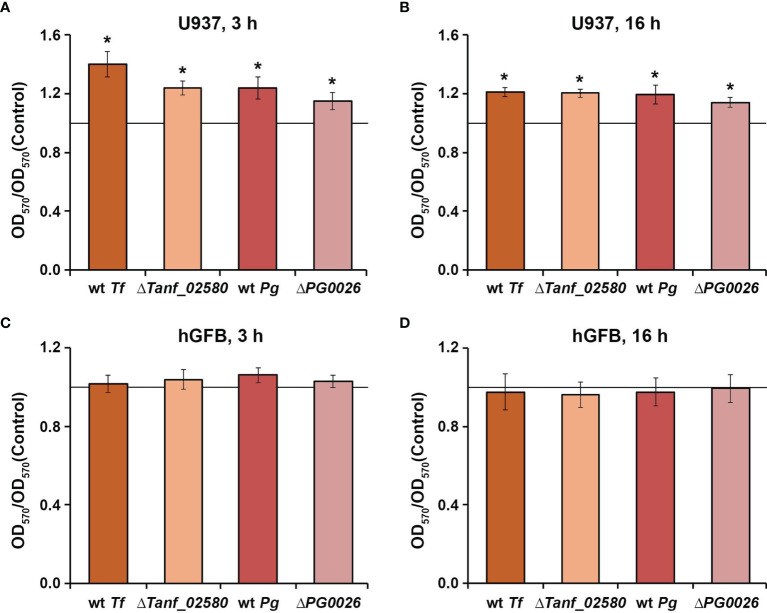
Viability of U937 macrophages **(A, B)** and hGFBs **(C, D)** upon stimulation with *T. forsythia* wild-type (wt *Tf*), Δ*Tanf_02580*, *P. gingivalis* wild-type (wt *Pg*), and Δ*PG0026* at MOI 50 for 3 h or 16 h. Cell viability was measured with the MTT assay. OD_570_ values were normalized to those measured for non-stimulated cells (control). Data are presented as mean ± s.e.m. of five independent experiments. * – Significantly different from control (a continues horizontal line), with *P* < 0.05.

All tested *T. forsythia* and *P. gingivalis* species promoted the viability of U937 macrophages, three hours and 16 hours post stimulation (**Figures 1A, B**). No differences in the viability of the macrophages were observed between challenges with wild-type species *versus* the corresponding T9SS-deficient mutant. In contrast, none of the tested bacteria had a significant influence on the viability of hGFBs after both three hours and 16 hours post stimulation (**Figures 1C, D**). Bacteria alone did not show a measurable reactivity with the MTT reagent under the chosen experimental conditions.

### Effect of *T. forsythia* Wild-Type and the Δ*Tanf_02580* Mutant on the Host Response of U937 Macrophages

**Figure 2:**
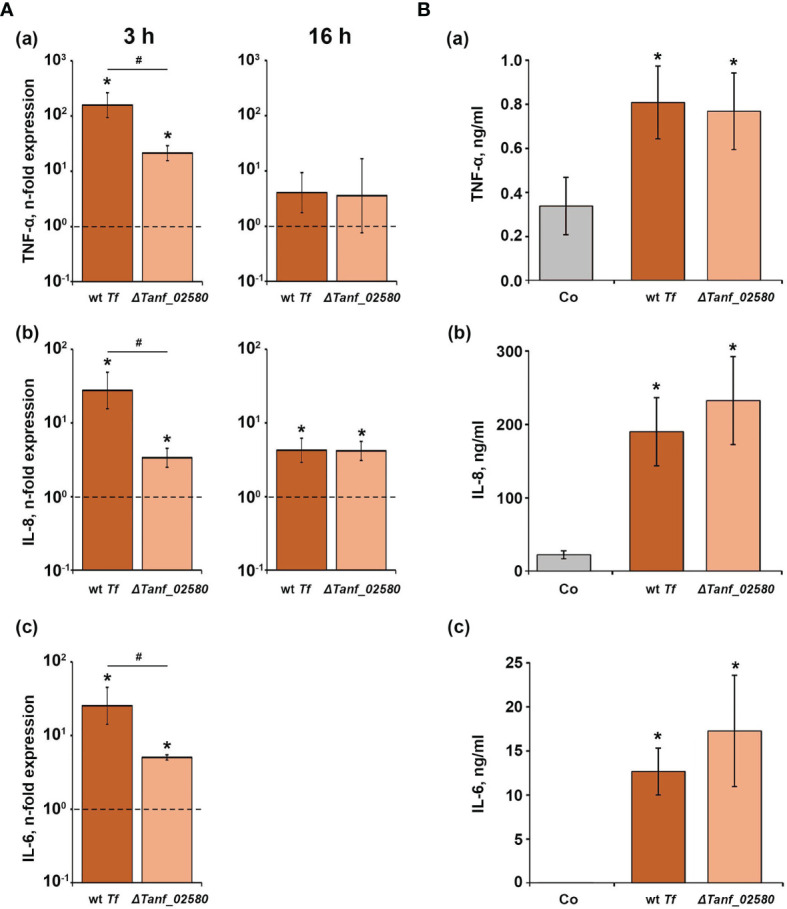
Comparison of the effects of *T. forsythia* wild-type (wt *Tf*) and T9SS signal peptidase mutant (Δ*Tanf_02580*) on the gene expression and protein production of TNF-α, IL-8, and IL-6 in U937 macrophages. **(A)** Macrophages were stimulated for 3 h (left panels) or 16 h (right panels) and the resulting expression of TNF-α **(a)**, IL-8 **(b)**, and IL-6 **(c)** was determined by qPCR. The *y*-axis shows n-fold expression of the target gene in relation to the unstimulated control (n-fold expression = 1, indicated by the dashed line) determined by the 2^-ΔΔCt^ method. IL-6 expression after 16 h was below the detection limit. **(B)** Macrophages were stimulated for 16 h and the resulting production of TNF-α **(a)**, IL-8 **(b)**, and IL-6 **(c)** was determined by ELISA. Non-stimulated cells served as a control (Co). All data are presented as mean ± s.e.m of six independent experiments. * – Significantly different from control, with *P* < 0.05. # – Significantly different between two *T. forsythia* species, with *P* < 0.05.

The effect of *T. forsythia* wild-type and Δ*Tanf_02580* on the gene expression levels of TNF-α, IL-6, and IL-8, and on the production of the corresponding proteins in U937 macrophages is shown in **Figure 2**. Both *T. forsythia* species increased the gene expression of all investigated inflammatory mediators (**Figure 2A**). Three hours post stimulation, *T. forsythia* wild-type induced significantly higher expression levels of all genes compared to the Δ*Tanf*_*02580* mutant. After 16 hours, neither *T. forsythia* strain had an effect on TNF-α gene expression, while both, wild-type and mutant, stimulated IL-8 gene expression by a similar degree. Gene expression of IL-6 after 16 hours was below the detection limit in the bacteria-treated groups. Sixteen hours post stimulation, both *T. forsythia* species increased the production of all investigated inflammatory mediators (**Figure 2B**), but no significant differences between *T. forsythia* wild-type and the T9SS signal peptidase mutant were observed.

### Effect of *T. forsythia* Wild-Type and the Δ*Tanf_02580* Mutant on the Host Response of Human Gingival Fibroblasts

**Figure 3:**
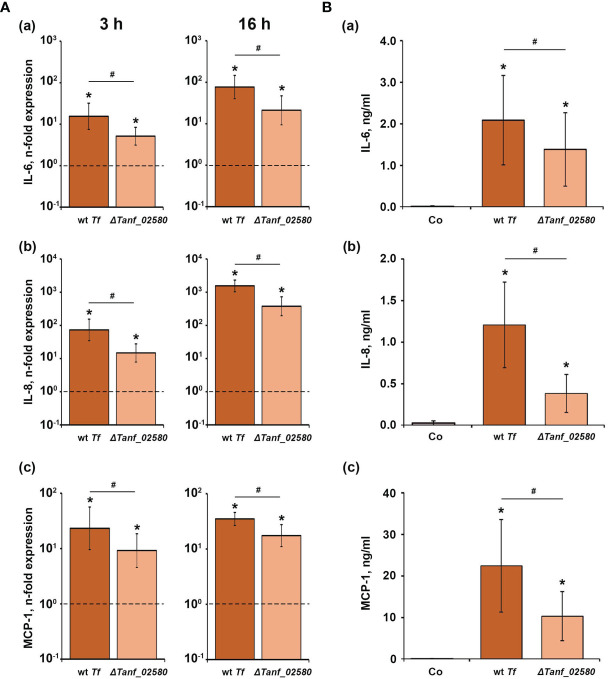
Comparison of the effects of *T. forsythia* wild-type (wt *Tf*) and T9SS signal peptidase mutant (Δ*Tanf_02580*) on the gene expression and protein production of TNF-α, IL-8, and IL-6 in hGFBs. **(A)** hGFBs were stimulated for 3 h (left panels) or 16 h (right panels) and the resulting expression of IL-6 **(a)**, IL-8 **(b)**, and MCP-1 **(c)** was determined by qPCR. The *y*-axis shows n-fold expression of the target gene in relation to the unstimulated control (n-fold expression = 1, indicated by the dashed line) determined by the 2^-ΔΔCt^ method. **(B)** hGFBs were stimulated for 16 h and the resulting production of IL-6 **(a)**, IL-8 **(b)**, and MCP-1 **(c)** was determined by ELISA. Non-stimulated cells served as a control (Co). All data are presented as mean ± s.e.m of six independent experiments. * – Significantly different from control, with *P* < 0.05. # – Significantly different between two *T. forsythia* species, with *P* < 0.05.

**Figure 3** shows the effect of *T. forsythia* wild-type and Δ*Tanf*_*02580* on the gene expression levels of IL-6, IL-8 and MCP-1, and the production of the corresponding proteins in hGFBs. Both *T. forsythia* species induced a significant increase in the gene expression of IL-6, IL-8, and MCP-1, three hours and 16 hours post stimulation. *T. forsythia* wild-type induced significantly higher gene expression levels than the Δ*Tanf_02580* mutant. Also, both *T. forsythia* species increased the production of all investigated cytokines compared to the control (**Figure 3B**), but, again, *T. forsythia* wild-type induced a significantly higher protein production than the T9SS signal peptidase mutant.

### Effect of *P. gingivalis* Wild-Type and the Δ*PG0026* Mutant on the Host Response of U937 Macrophages

**Figure 4:**
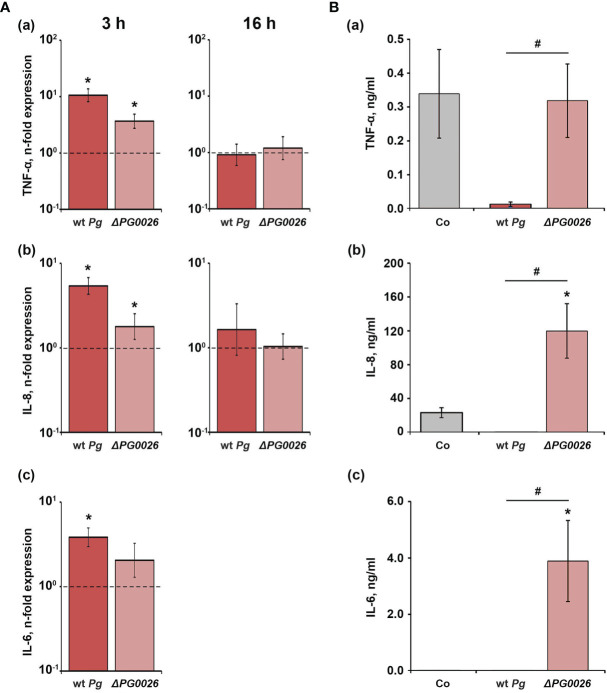
Comparison of the effects of *P. gingivalis* wild-type (wt *Pg*) and T9SS signal peptidase mutant (Δ*PG0026*) on the gene expression and protein production of TNF-α, IL-8, and IL-6 in U937 macrophages. **(A)** Macrophages were stimulated for 3 hours (left panels) or 16 hours (right panels) and the resulting expression of TNF-α **(a)**, IL-8 **(b)**, and IL-6 **(c)** was determined by qPCR. The *y*-axis shows n-fold expression of the target gene in relation to the unstimulated control (n-fold expression = 1, indicated by the dashed line) determined by the 2^-ΔΔCt^ method. IL-6 expression after 16 h was below the detection limit. **(B)** Macrophages were stimulated for 16 h and the resulting production of TNF-α **(a)**, IL-8 **(b)**, and IL-6 **(c)** was determined by ELISA. Non-stimulated cells served as a control (Co). All data are presented as mean ± s.e.m of six independent experiments. * – Significantly different from control, with *P* < 0.05. # – Significantly different between two *P. gingivalis* species, with *P* < 0.05.

The gene expression levels of TNF-α, IL-6, and IL-8 in U937 macrophages upon stimulation with *P. gingivalis* wild-type and the Δ*PG0026* mutant, and the levels of the corresponding proteins are shown in **Figure 4**. Three hours post stimulation, the expression of TNF-α, IL-6, and IL-8 was significantly increased in the *P. gingivalis* wild-type, whereas the Δ*PG0026* mutant significantly enhanced the expression of TNF-α and IL-8, but not of IL-6 (**Figure 4A**). No significant difference between the response of U937 macrophages to the two different *P. gingivalis* species was observed. Sixteen hours post stimulation, with both *P. gingivalis* species, the gene expression levels of TNF-α and IL-8 were similar to those in the unstimulated control. IL-6 expression after stimulation was below the detection limit (**Figure 4A**). After 16 hours, the levels of TNF-α and IL-8 in the conditioned media of *P. gingivalis* wild-type-treated macrophages were significantly lower than those in macrophages treated with the Δ*PG0026* mutant and the control group (**Figure 4B**). IL-6 protein in the conditioned media of *P. gingivalis* wild-type-treated macrophages was below the detection limit of the ELISA kit. Stimulation with the Δ*PG0026* mutant resulted in a significantly higher amount of IL-8 and IL-6 in the conditioned media compared to the control group, whereas TNF-α production was not affected.

### Effect of *P. gingivalis* Wild-Type and the Δ*PG0026* Mutant on the Host Response of Human Gingival Fibroblasts

**Figure 5:**
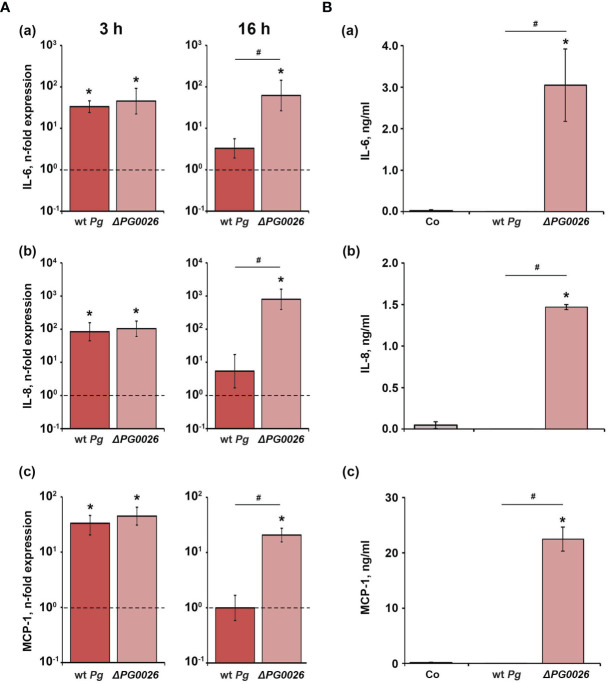
Comparison of the effects of *P. gingivalis* wild-type (wt *Pg*) and T9SS signal peptidase mutant (Δ*PG0026*) on the gene expression and protein production of TNF-α, IL-8, and IL-6 in hGFBs. **(A)** hGFBs were stimulated for 3 hours (left panels) or 16 hours (right panels) and the resulting expression of IL-6 **(a)**, IL-8 **(b)**, and MCP-1 **(c)** was determined by qPCR. The *y*-axis shows n-fold expression of the target gene in relation to the unstimulated control (n-fold expression = 1, indicated by the dashed line) determined by the 2^-ΔΔCt^ method. **(B)** hGFBs were stimulated for 16 h and the resulting production of IL-6 **(a)**, IL-8 **(b)**, and MCP-1 **(c)** was determined by ELISA. Non-stimulated cells served as a control (Co). All data are presented as mean ± s.e.m of six independent experiments. * – Significantly different from control, with *P* < 0.05. # – Significantly different between two *P. gingivalis* species, with *P* < 0.05.

The effect of *P. gingivalis* wild-type and Δ*PG0026* on the gene expression levels of IL-6, IL-8, and MCP-1 in hGFBs, and the levels of corresponding proteins in conditioned media are presented in **Figure 5**. Both *P. gingivalis* species induced a significant increase in the gene expression of IL-6, IL-8, and MCP-1 three hours post stimulation, but no difference between the two *P. gingivalis* species was observed. Sxiteen hours post stimulation, only the Δ*PG0026* mutant induced significantly higher gene expression levels of all investigated inflammatory mediators, which were also significantly higher than those in *P. gingivalis* wild-type-treated cells (**Figure 5A**). None of the proteins was detected in the conditioned media of *P. gingivalis* wild-type-stimulated hGFBs, whereas stimulation with the Δ*PG0026* mutant resulted in significantly higher amounts of all secreted inflammatory mediators compared to the unstimulated control (**Figure 5B**).

## Discussion

The periodontal pathogens *T. forsythia* and *P. gingivalis* have developed ingenious strategies to evade host immune clearance and to exploit their pathogenic potential (Amano et al., 2014). These bacteria direct a specific class of their proteins, namely those equipped with a CTD, to the T9SS-a translocon unique for *Bacteroidetes*–to display their harmful or self-protecting cargo at the cell surface or secrete it into the exterior environment (Veith et al., 2009; de Diego et al., 2016). Various virulence factors have been demonstrated to be targeted to the T9SS ensuring OM trafficking. In this study, we have analyzed if and how depletion of OM proteins channeled through this protein secretion system affects the immune response of human macrophages and hGFBs to *T. forsythia* and *P. gingivalis*.

By MS-shot-gun proteomics we first confirmed that the OM of *T. forsythia* and *P. gingivalis* T9SS mutants defective in secretion of CTD-protein due to deletion of the signal peptidase genes *Tanf*_*02580* and *PG0026*, respectively, were depleted from known CTD-proteins, whereas these were present in the parent strains. In the case of *T. forsythia*, this finding corroborates a previous analysis showing that the major CTD-proteins (TfsA and TfsB) of this bacterium forming the S-layer accumulated in the periplasm and the mutant lacked the S-layer (Tomek et al., 2014). Similarly, sortase mutants of *P. gingivalis* retained inactive gingipains in the periplasm (Mizgalska et al., 2021). Therefore, TfsA/TfsB and BspA surface antigen as well as the RgpA-, RgpB-, Kgp-gingipains from *P. gingivalis* served as leads for the assessment of the efficiency of T9SS shut-down in the signal peptidase mutants (**Tables 1** and **2**).

The viability of both human cell types included in this study upon bacterial infection was proven by the MTT assay, which is based on the measurements of mitochondrial cell activity (**Figure 1**). We found that at the tested MOI none of the bacterial species had a cytotoxic effect on the viability of macrophages and hGFBs (**Figure 1**). All bacteria induced an increase in the metabolic activity of macrophages, while this was not observed for hGFBs. The reason for this difference as well as the physiological importance of the increased viability of U937 macrophages after infection with *T. forsythia* and *P. gingivalis* is not entirely clear. Increased viability of macrophages might be due to the metabolic remodeling of these cells after bacterial infection (Fleetwood et al., 2017).

Shut-down of the T9SS in both *T. forsythia* and *P. gingivalis* altered the cytokine and chemokine response of host cells to these pathogens. The T9SS-deficient *T. forsythia* mutant Δ*Tanf_02580* generally induced a lower inflammatory response compared to the wild-type species in hGFBs as shown by qPCR and ELISA (**Figures 2** and **3**). A similar tendency was observed in U937 macrophages. As shown by proteomic analysis, shut-down of the T9SS in *T. forsythia* inhibits the secretion of several proteins, and particularly the S-layer proteins TfsA and TfsB (Tomek et al., 2014) as well as BspA, which have opposite effects on the host response. On the one hand, the *T. forsythia* S-layer was shown to delay the host response at the early phase of infection (Sekot et al., 2011); consequently, its absence might result in higher cytokine production by the host cells. On the other hand, BspA is known to be a strong TLR-2 agonist (Hajishengallis et al., 2002; Onishi et al., 2008). Both U937 and hGFBs express TLR-2 and produce inflammatory cytokines upon the stimulation with TLR-2 agonists (Greene et al., 2004; Behm et al., 2020) and, therefore, the impairment of BspA secretion results in a lower host response. Since we have observed a lower inflammatory host response to the Δ*Tanf*_*02580* mutant compared to the *T. forsythia* wild-type, we conclude that abolishing BspA OM translocation has a more profound effect on the secretion of the tested inflammatory mediators. Quantitative differences between macrophages and hGFBs might be explained by the generally different responses of these cell types to various pathogen-associated molecular patterns. Particularly, our previous studies showed that the activation of the TLR-2 pathway induces a strong response in hGFBs and periodontal ligament cells, which is markedly higher than that induced by the TLR-4 agonist lipopolysaccharide (Andrukhov et al., 2016; Blufstein et al., 2019). Therefore, elimination of the secretion of the TLR-2 agonist BspA in the Δ*Tanf_02580* mutant would strongly diminish the response of this cell type to *T. forsythia*.

Compared to *T. forsythia*, the deletion of the T9SS signal peptidase in *P. gingivalis* had a qualitatively strikingly different effect on the host response. *P. gingivalis* wild-type induced generally higher gene expression of TNF-α, IL-8, and IL-6 in U937 macrophages three hours post stimulation compared to the Δ*PG0026* mutant. However, the content of all investigated proteins in conditioned media was not increased after stimulation with *P. gingivalis* wild-type. Moreover, the levels of most of the investigated proteins after 3 hours were below the detection limit of the ELISA kit. In contrast, after stimulation with the Δ*PG0026* mutant, a significantly increased amount of IL-6 and IL-8 was detected in the supernatant of U937 macrophages. This might be explained by the high activity of the *P. gingivalis* gingipains, which in fact are major virulence factors of this bacterium (Genco et al., 1999; Bao et al., 2014; O’Brien-Simpson et al., 2016). Gingipains have been shown to activate the host response by inducing secretion of IL-6 by oral epithelial cells (Lourbakos et al., 2001), of IL-8 by gingival fibroblasts (Oido-Mori et al., 2001), and of IL-6, IL-8 and MCP-1 in monocytic cells (Uehara et al., 2008). This is reflected by the slightly lower gene expression levels observed after stimulation with the T9SS-deficient mutant Δ*PG0026* when compared to the wild-type. On the other hand, gingipains are potent in the proteolytic cleavage of various host proteins, including inflammatory mediators (Yun et al., 1999; Yun et al., 2002; Tam et al., 2009). Thus, the secretion of gingipains by *P. gingivalis* wild-type has two consequences for the host cells’ response; they might stimulate the gene expression and at the same time degrade the secreted proteins. This assumption might explain the qualitative differences in gene and protein expression in the response of U937 macrophages to two *P. gingivalis* species.

In hGFBs, the effects of wild-type and T9SS-deficient *P. gingivalis* were slightly different compared to U937 macrophages (**Figures 4**, **5**). After 3 hours, the response of hGFBs to both species was similar on the gene expression level, whereas 16 hours post stimulation, the response to the T9SS-deficient mutant was markedly higher than that to *P. gingivalis* wild-type. Gingipain activity of the wild-type might also explain this difference. The response to bacterial pathogens in hGFBs might be increased by autocrine mechanisms mediated by IL-1β and TNF-α production (Naruishi and Nagata, 2018). This autocrine loop might be disrupted by gingipains, which would then result in lower gene expression levels upon the stimulation with *P. gingivalis* wild-type. Furthermore, gingipains have been shown to facilitate *P. gingivalis* cell adhesion and invasion (Chen and Duncan, 2004; Boisvert and Duncan, 2008; Fitzpatrick et al., 2009). In gingival epithelial cells, intracellular *P. gingivalis* can suppress the production of IL-8, interferon-γ induced protein 10 and TLR-2 and inhibit apoptosis (Hajishengallis and Lamont, 2014). Blocking gingipain secretion in the T9SS-deficient mutant might result in a reduced ability to invade hGFBs and, therefore, higher gene expression levels can be observed after stimulation with this strain after 16 hours when compared to cells infected with the wild-type.

When comparing the response of U937 macrophages and hGFBs to the different bacterial species, the different intrinsic properties of these cell types need to be considered. Macrophages are immune cells and their response to bacteria is transient - recovery follows the initial increase in the gene expression to the initial levels within 24 hours (Nau et al., 2002). In contrast, hGFBs are assumed to contribute to a sustained inflammation and do not exhibit a tolerance state after prolonged stimulation with bacterial components (Ara et al., 2009; Blufstein et al., 2018).

## Conclusions

We demonstrated that the shut-down of OM translocation of CTD-proteins *via* the T9SS in *T. forsythia* and *P. gingivalis* causes an alteration of the host immune response to these pathogens, Considering our data and those from the literature, it is conceivable to assume that the decreased host response to the *T. forsythia* T9SS-signal peptidase mutant and the drastically changed host response to the *P. gingivalis* mutant in comparison to the respective parent strain are largely due to the impaired secretion of BspA and the S-layer proteins in the *T. forsythia* T9SS mutant and the activity of the gingipains in the *P. gingivalis* T9SS mutant, respectively. Blocking gingipain secretion in *P. gingivalis* seems to have a dual effect, associated with both activation and inactivation of the host response (Imamura et al., 2003).

The abrogation of the T9SS secretion system in periodontal pathogens can have both pro- and anti-inflammatory effects and, therefore, the contribution of the T9SS to the host-microbiome interaction in the oral cavity needs further clarification. In a subcutaneous chamber model of infection in mice, the shut-down of the T9SS in *P. gingiva*lis resulted in lower inflammation and decreased systemic dissemination of infection (Benedyk et al., 2019). However, the overall role of the T9SS in human periodontitis needs to be investigated in future basic and clinical studies. This might unravel future strategies for the treatment of periodontitis and/or prophylaxis of periodontitis.

## Data Availability Statement

The original contributions presented in the study are included in the article/**Supplementary Material**. Further inquiries can be directed to the corresponding authors.

## Ethics Statement

The procedure for the isolation of hGFBs from the gingival tissue was approved by the Ethic Committee of the Medical University of Vienna (EK 1694/2015, extended in 2019). All patients gave their written consent. The patients/participants provided their written informed consent to participate in this study.

## Author Contributions

Conceptualization, CS and OA. Methodology, CS and OA. Validation, CS, OA, SB, and CG-G. Formal analysis, MB, MT, SB, CG-G, PN, CS, and OA. Investigation, MB, MT, SB, CG-G, and PN. Writing—original draft preparation, MB, OA, and CS. Writing—review and editing, MT, SB, JP, OA, and CS. Supervision, CS and OA. Funding acquisition, CS. All authors have read and agreed to the published version of the manuscript.

## Funding

This research was funded by Austrian Science Fund FWF, projects P24317-B22 and P33618-B22 (to CS) and the FWF Doctoral Programme “BioToP – Biomolecular Technology of Proteins” W1224. OA was supported by Austrian Science Fund FWF, project P29440. JP was supported by NIH/NIDCR grants DE026280 and DE030939. Open Access Funding by the Austrian Science Fund (FWF). The funders had no role in the design of the study; in the collection, analyses, or interpretation of data; in the writing of the manuscript, or in the decision to publish the results.

## Conflict of Interest

The authors declare that the research was conducted in the absence of any commercial or financial relationships that could be construed as a potential conflict of interest.

## Publisher’s Note

All claims expressed in this article are solely those of the authors and do not necessarily represent those of their affiliated organizations, or those of the publisher, the editors and the reviewers. Any product that may be evaluated in this article, or claim that may be made by its manufacturer, is not guaranteed or endorsed by the publisher.

## References

[B1] Amano A. Chen C. Honma K. Li C. Settem R. P. Sharma A. (2014). Genetic Characteristics and Pathogenic Mechanisms of Periodontal Pathogens. Adv. Dent. Res. 26, 15–22. doi: 10.1177/0022034514526237 24736700 PMC6636228

[B2] Andrukhov O. Andrukhova O. Ozdemir B. Haririan H. Muller-Kern M. Moritz A.. (2016). Soluble CD14 Enhances the Response of Periodontal Ligament Stem Cells to *P. gingivalis* Lipopolysaccharide. PloS One 11, e0160848. doi: 10.1371/journal.pone.0160848 27504628 PMC4978456

[B3] Ara T. Kurata K. Hirai K. Uchihashi T. Uematsu T. Imamura Y.. (2009). Human Gingival Fibroblasts are Critical in Sustaining Inflammation in Periodontal Disease. J. Periodontal Res. 44, 21–27. doi: 10.1111/j.1600-0765.2007.01041.x 19515019

[B4] Baggiolini M. Clark-Lewis I. (1992). Interleukin-8, a Chemotactic and Inflammatory Cytokine. FEBS Lett. 307, 97–101. doi: 10.1016/0014-5793(92)80909-Z 1639201

[B5] Bao K. Belibasakis G. N. Thurnheer T. Aduse-Opoku J. Curtis M. A. Bostanci N. (2014). Role of *Porphyromonas gingivalis* Gingipains in Multi-Species Biofilm Formation. BMC Microbiol. 14, 258. doi: 10.1186/s12866-014-0258-7 25270662 PMC4189655

[B6] Behm C. Blufstein A. Abhari S. Y. Koch C. Gahn J. Schäffer C.. (2020). Response of Human Mesenchymal Stromal Cells From Periodontal Tissue to LPS Depends on the Purity But Not on the LPS Source. Mediators Inflamm. 2020, 8704896. doi: 10.1155/2020/8704896 32714091 PMC7352132

[B7] Behm C. Blufstein A. Gahn J. Noroozkhan N. Moritz A. Rausch-Fan X.. (2019). Soluble CD14 Enhances the Response of Periodontal Ligament Stem Cells to Toll-Like Receptor 2 Agonists. Mediators Inflamm. 2019, 8127301. doi: 10.1155/2019/8127301 31178663 PMC6507176

[B8] Benedyk M. Marczyk A. Chruscicka B. (2019). Type IX Secretion System is Pivotal for Expression of Gingipain-Associated Virulence of *Porphyromonas gingivalis* . Mol. Oral. Microbiol. 34, 237–244. doi: 10.1111/omi.12268 31432617

[B9] Blufstein A. Behm C. Gahn J. Uitz O. Naumovska I. Moritz A.. (2019). Synergistic Effects Triggered by Simultaneous Toll-Like Receptor-2 and -3 Activation in Human Periodontal Ligament Stem Cells. J. Periodontol 90, 1190–1201. doi: 10.1002/JPER.19-0005 31049957 PMC6852053

[B10] Blufstein A. Behm C. Nguyen P. Q. Rausch-Fan X. Andrukhov O. (2018). Human Periodontal Ligament Cells Exhibit No Endotoxin Tolerance Upon Stimulation With *Porphyromonas gingivalis* Lipopolysaccharide. J. Periodontal Res. 53, 589–597. doi: 10.1111/jre.12549 29582430 PMC6055822

[B11] Boisvert H. Duncan M. J. (2008). Clathrin-Dependent Entry of a Gingipain Adhesin Peptide and *Porphyromonas gingivalis* Into Host Cells. Cell Microbiol. 10, 2538–2552. doi: 10.1111/j.1462-5822.2008.01228.x 18717820 PMC3016922

[B12] Bradford M. M. (1976). A Rapid and Sensitive Method for the Quantitation of Microgram Quantities of Protein Utilizing the Principle of Protein-Dye Binding. Anal. Biochem. 72, 248–254. doi: 10.1006/abio.1976.9999 942051

[B13] Cekici A. Kantarci A. Hasturk H. Van Dyke T. E. (2014). Inflammatory and Immune Pathways in the Pathogenesis of Periodontal Disease. Periodontol 2000 64, 57–80. doi: 10.1111/prd.12002 24320956 PMC4500791

[B14] Charlie-Silva I. Klein A. Gomes J. M. M. Prado E. J. R. Moraes A. C. Eto S. F.. (2019). Acute-Phase Proteins During Inflammatory Reaction by Bacterial Infection: Fish-Model. Sci. Rep. 9, 4776. doi: 10.1038/s41598-019-41312-z 30886242 PMC6423045

[B15] Chen T. Duncan M. J. (2004). Gingipain Adhesin Domains Mediate *Porphyromonas gingivalis* Adherence to Epithelial Cells. Microb. Pathogen 36, 205–209. doi: 10.1016/j.micpath.2003.12.001 15001226

[B16] de Diego I. Ksiazek M. Mizgalska D. Koneru L. Golik P. Szmigielski B.. (2016). The Outer-Membrane Export Signal of *Porphyromonas gingivalis* Type IX Secretion System (T9SS) is a Conserved C-Terminal Beta-Sandwich Domain. Sci. Rep. 6, 23123. doi: 10.1038/srep23123 27005013 PMC4804311

[B17] Deshmane S. L. Kremlev S. Amini S. Sawaya B. E. (2009). Monocyte Chemoattractant Protein-1 (MCP-1): An Overview. J. Interfern Cytokine Res. 29, 313–326. doi: 10.1089/jir.2008.0027 PMC2755091 19441883

[B18] Fitzpatrick R. E. Wijeyewickrema L. C. Pike R. N. (2009). The Gingipains: Scissors and Glue of the Periodontal Pathogen, *Porphyromonas gingivalis* . Fut Microbiol. 4, 471–487. doi: 10.2217/Fmb.09.18 19416015

[B19] Fleetwood A. J. Lee M. K. S. Singleton W. Achuthan A. Lee M. C. O’Brien-Simpson N. M.. (2017). Metabolic Remodeling, Inflammasome Activation, and Pyroptosis in Macrophages Stimulated by *Porphyromonas gingivalis* and Its Outer Membrane Vesicles. Front. Cell Infect. Microbiol. 7, 351. doi: 10.3389/fcimb.2017.00351 28824884 PMC5543041

[B20] Friedrich V. Gruber C. Nimeth I. Pabinger S. Sekot G. Posch G.. (2015). Outer Membrane Vesicles of *Tannerella forsythia*: Biogenesis, Composition, and Virulence. Mol. Oral. Microbiol. 30, 451–473. doi: 10.1111/omi.12104 25953484 PMC4604654

[B21] Genco C. A. Potempa J. Mikolajczyk-Pawlinska J. Travis J. (1999). Role of Gingipains R in the Pathogenesis of *Porphyromonas gingivalis*-Mediated Periodontal Disease. Clin. Infect. Dis. 28, 456–465. doi: 10.1086/515156 10194062

[B22] Gorasia D. G. Glew M. D. Veith P. D. Reynolds E. C. (2020). Quantitative Proteomic Analysis of the Type IX Secretion System Mutants in *Porphyromonas gingivalis* . Mol. Oral. Microbiol. 35, 78–84. doi: 10.1111/omi.12283 32040252

[B23] Greene C. M. McElvaney N. G. O’Neill S. J. Taggart C. C. (2004). Secretory Leucoprotease Inhibitor Impairs Toll-Like Receptor 2- and 4-Mediated Responses in Monocytic Cells. Infect. Immun. 72, 3684–3687. doi: 10.1128/iai.72.6.3684-3687.2004 15155685 PMC415654

[B24] Griffen A. L. Beall C. J. Campbell J. H. Firestone N. D. Kumar P. S. Yang Z. K.. (2012). Distinct and Complex Bacterial Profiles in Human Periodontitis and Health Revealed by 16S Pyrosequencing. ISME J. 6, 1176–1185. doi: 10.1038/ismej.2011.191 22170420 PMC3358035

[B25] Gundry R. L. White M. Y. Murray C. I. Kane L. A. Fu Q. Stanley B. A.. (2009). Preparation of Proteins and Peptides for Mass Spectrometry Analysis in a Bottom-Up Proteomics Workflow. Curr. Prot Mol. Biol. Chapter 10, Unit10.25–Unit10.25. doi: 10.1002/0471142727.mb1025s88 PMC2905857 19816929

[B26] Hajishengallis G. Lamont R. J. (2012). Beyond the Red Complex and Into More Complexity: The Polymicrobial Synergy and Dysbiosis (PSD) Model of Periodontal Disease Etiology. Mol. Oral. Microbiol. 27, 409–419. doi: 10.1111/j.2041-1014.2012.00663.x 23134607 PMC3653317

[B27] Hajishengallis G. Lamont R. J. (2014). Breaking Bad: Manipulation of the Host Response by *Porphyromonas gingivalis* . Eur. J. Immunol. 44, 328–338. doi: 10.1002/eji.201344202 24338806 PMC3925422

[B28] Hajishengallis G. Martin M. Sojar H. T. Sharma A. Schifferle R. E. DeNardin E.. (2002). Dependence of Bacterial Protein Adhesins on Toll-Like Receptors for Proinflammatory Cytokine Induction. Clin. Diagn. Lab. Immunol. 9, 403–411. doi: 10.1128/cdli.9.2.403-411.2002 11874886 PMC119939

[B29] Holt S. C. Ebersole J. L. (2005). *Porphyromonas gingivalis*, *Treponema denticola*, and *Tannerella forsythia*: The “Red Complex”, a Prototype Polybacterial Pathogenic Consortium in Periodontitis. Periodontol 2000 38, 72–122. doi: 10.1111/j.1600-0757.2005.00113.x 15853938

[B30] Imamura T. Travis J. Potempa J. (2003). The Biphasic Virulence Activities of Gingipains: Activation and Inactivation of Host Proteins. Curr. Protein Pept. Sci. 4, 443–450. doi: 10.2174/1389203033487027 14683429

[B31] Komatsuzawa H. Asakawa R. Kawai T. Ochiai K. Fujiwara T. Taubman M. A.. (2002). Identification of Six Major Outer Membrane Proteins From *Actinobacillus actinomycetemcomitans* . Gene 288, 195–201. doi: 10.1016/s0378-1119(02)00500-0 12034509

[B32] Koneru L. Ksiazek M. Waligorska I. Straczek A. Lukasik M. Madej M.. (2017). Mirolysin, a LysargiNase From *Tannerella forsythia*, Proteolytically Inactivates the Human Cathelicidin, LL-37. Biol. Chem. 398, 395–409. doi: 10.1515/hsz-2016-0267 27997347 PMC5478484

[B33] Ksiazek M. Mizgalska D. Eick S. Thøgersen I. B. Enghild J. J. Potempa J. (2015). KLIKK Proteases of *Tannerella forsythia*: Putative Virulence Factors With a Unique Domain Structure. Front. Microbiol. 6, 312. doi: 10.3389/fmicb.2015.00312 25954253 PMC4404884

[B34] Laemmli U. K. (1970). Cleavage of Structural Proteins During the Assembly of the Head of Bacteriophage T4. Nature 227, 680–685. doi: 10.1038/227680a0 5432063

[B35] Lasica A. M. Goulas T. Mizgalska D. Zhou X. de Diego I. Ksiazek M.. (2016). Structural and Functional Probing of PorZ, an Essential Bacterial Surface Component of the Type-IX Secretion System of Human Oral-Microbiomic *Porphyromonas gingivalis* . Sci. Rep. 6, 37708. doi: 10.1038/srep37708 27883039 PMC5121618

[B36] Lasica A. M. Ksiazek M. Madej M. Potempa J. (2017). The Type IX Secretion System (T9SS): Highlights and Recent Insights Into its Structure and Function. Front. Cell Infect. Microbiol. 7, 215. doi: 10.3389/fcimb.2017.00215 28603700 PMC5445135

[B37] Lauber F. Deme J. C. Lea S. M. Berks B. C. (2018). Type 9 Secretion System Structures Reveal a New Protein Transport Mechanism. Nature 564, 77–82. doi: 10.1038/s41586-018-0693-y 30405243 PMC6927815

[B38] Lee S. W. Sabet M. Um H. S. Yang J. Kim H. C. Zhu W. (2006). Identification and Characterization of the Genes Encoding a Unique Surface (S-) Layer of *Tannerella forsythia* . Gene 371, 102–111. doi: 10.1016/j.gene.2005.11.027 16488557

[B39] Lourbakos A. Potempa J. Travis J. D’Andrea M. R. Andrade-Gordon P. Santulli R.. (2001). Arginine-Specific Protease From *Porphyromonas gingivalis* Activates Protease-Activated Receptors on Human Oral Epithelial Cells and Induces Interleukin-6 Secretion. Infect. Immun. 69, 5121–5130. doi: 10.1128/IAI.69.8.5121-5130.2001 11447194 PMC98608

[B40] Marsh and Zaura (2011). Dental biofilm: ecological interactions in health and disease. J. Clin. Periodontol. 44 (Suppl 18), S12–S22. doi: 10.1111/jcpe.12679 28266111

[B41] Mishima E. Sharma A. (2011). *Tannerella forsythia* Invasion in Oral Epithelial Cells Requires Phosphoinositide 3-Kinase Activation and Clathrin-Mediated Endocytosis. Microbiology 157, 2382–2391. doi: 10.1099/mic.0.048975-0 21622527 PMC3167883

[B42] Mizgalska D. Goulas T. Rodríguez-Banqueri A. Veillard F. Madej M. Małecka E.. (2021). Intermolecular Latency Regulates the Essential C-Terminal Signal Peptidase and Sortase of the *Porphyromonas gingivalis* Type-IX Secretion System. Proc. Natl. Acad. Sci. U.S.A. 118. doi: 10.1073/pnas.2103573118 PMC8501833 34593635

[B43] Murakami Y. Higuchi N. Nakamura H. Yoshimura F. Oppenheim F. G. (2002). *Bacteroides forsythus* Hemagglutinin is Inhibited by *N-*Acetylneuraminyllactose. Oral. Microbiol. Immunol. 17, 125–128. doi: 10.1046/j.0902-0055.2001.00093.x 11929561

[B44] Naruishi K. Nagata T. (2018). Biological Effects of Interleukin-6 on Gingival Fibroblasts: Cytokine Regulation in Periodontitis. J. Cell Physiol. 233, 6393–6400. doi: 10.1002/jcp.26521 29574949 PMC13482493

[B45] Nau G. J. Richmond J. F. Schlesinger A. Jennings E. G. Lander E. S. Young R. A. (2002). Human Macrophage Activation Programs Induced by Bacterial Pathogens. Proc. Natl. Acad. Sci. U.S.A. 99, 1503–1508. doi: 10.1073/pnas.022649799 11805289 PMC122220

[B46] Nguyen K. A. Travis J. Potempa J. (2007). Does the Importance of the C-Terminal Residues in the Maturation of RgpB From *Porphyromonas gingivalis* Reveal a Novel Mechanism for Protein Export in a Subgroup of Gram-Negative Bacteria? J. Bacteriol 189, 833–843. doi: 10.1128/jb.01530-06 17142394 PMC1797278

[B47] O’Brien-Simpson N. M. Holden J. A. Lenzo J. C. Tan Y. Brammar G. C. Walsh K. A.. (2016). A Therapeutic *Porphyromonas gingivalis* Gingipain Vaccine Induces Neutralising IgG1 Antibodies That Protect Against Experimental Periodontitis. NPJ Vaccines 1, 16022. doi: 10.1038/npjvaccines.2016.22 29263860 PMC5707886

[B48] Oido-Mori M. Rezzonico R. Wang P. L. Kowashi Y. Dayer J. M. Baehni P. C.. (2001). *Porphyromonas gingivalis* Gingipain-R Enhances Interleukin-8 But Decreases Gamma Interferon-Inducible Protein 10 Production by Human Gingival Fibroblasts in Response to T-Cell Contact. Infect. Immun. 69, 4493–4501. doi: 10.1128/Iai.69.7.4493-4501.2001 11401991 PMC98524

[B49] Olsen I. Hajishengallis G. (2016). Major Neutrophil Functions Subverted by *Porphyromonas gingivalis* . J. Oral. Microbiol. 8, 30936. doi: 10.3402/jom.v8.30936 26993626 PMC4799392

[B50] Onishi S. Honma K. Liang S. Stathopoulou P. Kinane D. Hajishengallis G.. (2008). Toll-Like Receptor 2-Mediated Interleukin-8 Expression in Gingival Epithelial Cells by the *Tannerella forsythia* Leucine-Rich Repeat Protein BspA. Infect. Immun. 76, 198–205. doi: 10.1128/iai.01139-07 17967853 PMC2223669

[B51] Posch G. Sekot G. Friedrich V. Megson Z. A. Koerdt A. Messner P.. (2012). Glycobiology Aspects of the Periodontal Pathogen *Tannerella forsythia* . Biomolecules 2, 467–482. doi: 10.3390/biom2040467 24970146 PMC4030854

[B52] Rangarajan M. Smith S. J. U S. Curtis M. A. (1997). Biochemical Characterization of the Arginine-Specific Proteases of *Porphyromonas gingivalis* W50 Suggests a Common Precursor. Biochem. J. 323 (Pt 3), 701–709. doi: 10.1042/bj3230701 9169603 PMC1218373

[B53] Sabet M. Lee S.-W. Nauman R. K. Sims T. Um H.-S. (2003). The Surface (S-) Layer is a Virulence Factor of *Bacteroides forsythus* . Microbiology 149, 3617–3627. doi: 10.1099/mic.0.26535-0 14663093

[B54] Sakakibara J. Nagano K. Murakami Y. Higuchi N. Nakamura H. Shimozato K.. (2007). Loss of Adherence Ability to Human Gingival Epithelial Cells in S-Layer Protein-Deficient Mutants of *Tannerella forsythensis* . Microbiology 153, 866–876. doi: 10.1099/mic.0.29275-0 17322207

[B55] Sato K. Yukitake H. Narita Y. Shoji M. Naito M. Nakayama K. (2013). Identification of *Porphyromonas gingivalis* Proteins Secreted by the Por Secretion System. FEMS Microbiol. Lett. 338, 68–76. doi: 10.1111/1574-6968.12028 23075153

[B56] Seers C. A. Slakeski N. Veith P. D. Nikolof T. Chen Y. Y. Dashper S. G.. (2006). The RgpB C-Terminal Domain has a Role in Attachment of RgpB to the Outer Membrane and Belongs to a Novel C-Terminal-Domain Family Found in *Porphyromonas gingivalis* . J. Bacteriol 188, 6376–6386. doi: 10.1128/jb.00731-06 16923905 PMC1595369

[B57] Sekot G. Posch G. Messner P. Matejka M. Rausch-Fan X. Andrukhov O.. (2011). Potential of the *Tannerella forsythia* S-Layer to Delay the Immune Response. J. Dental Res. 90, 109–114. doi: 10.1177/0022034510384622 PMC4382719 20929722

[B58] Sharma A. Sojar H. T. Glurich I. Honma K. Kuramitsu H. K. Genco R. J. (1998). Cloning, Expression, and Sequencing of a Cell Surface Antigen Containing a Leucine-Rich Repeat Motif From *Bacteroides forsythus* ATCC 43037. Infect. Immun. 66, 5703–5710. doi: 10.1128/IAI.66.12.5703-5710.1998 9826345 PMC108721

[B59] Shimotahira N. Oogai Y. Kawada-Matsuo M. Yamada S. Fukutsuji K. Nagano K.. (2013). The Surface Layer of *Tannerella forsythia* Contributes to Serum Resistance and Oral Bacterial Coaggregation. Infect. Immun. 81, 1198–1206. doi: 10.1128/IAI.00983-12 23357386 PMC3639587

[B60] Sochalska M. Potempa J. (2017). Manipulation of Neutrophils by *Porphyromonas gingivalis* in the Development of Periodontitis. Front. Cell Infect. Microbiol. 7, 197. doi: 10.3389/fcimb.2017.00197 28589098 PMC5440471

[B61] Socransky S. S. Haffajee A. D. (2005). Periodontal Microbial Ecology. Periodontol 2000 38, 135–187. doi: 10.1111/j.1600-0757.2005.00107.x 15853940

[B62] Stathopoulou P. G. Benakanakere M. R. Galicia J. C. Kinane D. F. (2009). The Host Cytokine Response to *Porphyromonas gingivalis* is Modified by Gingipains. Oral. Microbiol. Immunol. 24, 11–17. doi: 10.1111/j.1399-302X.2008.00467.x 19121064 PMC2717190

[B63] Stathopoulou P. G. Benakanakere M. R. Galicia J. C. Kinane D. F. (2010). Epithelial Cell Pro-Inflammatory Cytokine Response Differs Across Dental Plaque Bacterial Species. J. Clin. Periodontol 37, 24–29. doi: 10.1111/j.1600-051X.2009.01505.x 20096064 PMC2900159

[B64] Tada H. Shimizu T. Matsushita K. Takada H. (2017). *Porphyromonas gingivalis-*Induced IL-33 Down-Regulates hCAP-18/LL-37 Production in Human Gingival Epithelial Cells. BioMed. Res. 38, 167–173. doi: 10.2220/biomedres.38.167 28637951

[B65] Tam V. O’Brien-Simpson N. M. Chen Y. Y. Sanderson C. J. Kinnear B. Reynolds E. C. (2009). The RgpA-Kgp Proteinase-Adhesin Complexes of *Porphyromonas gingivalis* Inactivate the Th2 Cytokines Interleukin-4 and Interleukin-5. Infect. Immun. 77, 1451–1458. doi: 10.1128/IAI.01377-08 19168731 PMC2663161

[B66] Tomek M. B. Neumann L. Nimeth I. Koerdt A. Andesner P. Messner P.. (2014). The S-Layer Proteins of *Tannerella forsythia* are Secreted *via* a Type IX Secretion System That is Decoupled From Protein *O-*Glycosylation. Mol. Oral. Microbiol. 29, 307–320. doi: 10.1111/omi.12062 24943676 PMC4232474

[B67] Uehara A. Imamura T. Potempa J. Travis J. Takada H. (2008). Gingipains From *Porphyromonas gingivalis* Synergistically Induce the Production of Proinflammatory Cytokines Through Protease-Activated Receptors With Toll-Like Receptor and NOD1/2 Ligands in Human Monocytic Cells. Cell Microbiol. 10, 1181–1189. doi: 10.1111/j.1462-5822.2008.01119.x 18182086

[B68] Veith P. D. O’Brien-Simpson N. M. Tan Y. Djatmiko D. C. Dashper S. G. Reynolds E. C. (2009). Outer Membrane Proteome and Antigens of *Tannerella forsythia* . J. Proteome Res. 8, 4279–4292. doi: 10.1021/pr900372c 19663511

[B69] Veith P. D. Shoji M. O’Hair R. A. J. Leeming M. G. Nie S. Glew M. D.. (2020). Type IX Secretion System Cargo Proteins are Glycosylated at the C-Terminus With a Novel Linking Sugar of the Wbp/Vim Pathway. mBio 11. doi: 10.1128/mBio.01497-20 PMC7468200 32873758

[B70] Vistica D. T. Skehan P. Scudiero D. Monks A. Pittman A. Boyd M. R. (1991). Tetrazolium-Based Assays for Cellular Viability: A Critical Examination of Selected Parameters Affecting Formazan Production. Cancer Res. 51, 2515–2520. 2021931

[B71] Wilensky A. Tzach-Nahman R. Potempa J. Shapira L. Nussbaum G. (2015). *Porphyromonas gingivalis* Gingipains Selectively Reduce CD14 Expression, Leading to Macrophage Hyporesponsiveness to Bacterial Infection. J. Innate Immun. 7, 127–135. doi: 10.1159/000365970 25228314 PMC4348206

[B72] Yun P. L. W. DeCarlo A. A. Collyer C. Hunter N. (2002). Modulation of an Interleukin-12 and Gamma Interferon Synergistic Feedback Regulatory Cycle of T-Cell and Monocyte Cocultures by *Porphyromonas gingivalis* Lipopolysaccharide in the Absence or Presence of Cysteine Proteinases. Infect. Immun. 70, 5695–5705. doi: 10.1128/Iai.70.10.5695-5705.2002 12228299 PMC128344

[B73] Yun P. L. W. DeCarlo A. A. Hunter N. (1999). Modulation of Major Histocompatibility Complex Protein Expression by Human Gamma Interferon Mediated by Cysteine Proteinase-Adhesin Polyproteins of *Porphyromonas gingivalis* . Infect. Immun. 67, 2986–2995. doi: 10.1128/Iai.67.6.2986-2995.1999 10338509 PMC96610

