# Supplementary material for: Shut-Down of Type IX Protein Secretion Alters the Host Immune Response to Tannerella forsythia and Porphyromonas gingivalis
Source: Front Cell Infect Microbiol. 2022 Feb 10;12:835509. doi: 10.3389/fcimb.2022.835509 (PMC8869499; doi:10.3389/fcimb.2022.835509)
Supplement: Supplementary file 1 [file DataSheet_1.pdf]

frontiers

Supplementary Material

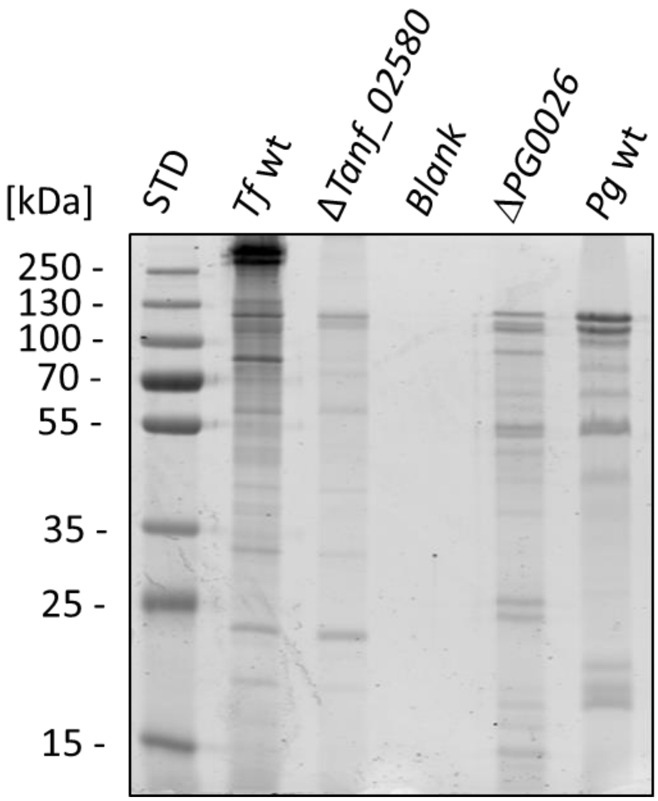

Supplementary Figure 1. SDS-PAGE analysis of outer membrane OM) preparations from *T. forsythia* wild-type (*Tf* wt), Δ*TF0955* mutant, Δ*PG0026* and *P. gingivalis* wild-type (*Pg* wt) upon protein staining with Coomassie Brilliant Blue. PageRuler™ Plus Prestained Protein Ladder (Thermo Scientific). 5 µg of total OM proteins were loaded per lane.
